# Understanding the Impact of Long COVID on the Lives of Thai University Students

**DOI:** 10.3390/ijerph23060687

**Published:** 2026-05-22

**Authors:** Valainipha Habuddha, Nitita Piya-amornphan

**Affiliations:** 1 Movement Science and Exercise Research Center-Walailak University (MoveSE-WU), Walailak University, Nakhon Si Thammarat 80160, Thailand; hvalaini@wu.ac.th; 2 Department of Physical Therapy, School of Allied Health Sciences, Walailak University, Nakhon Si Thammarat 80160, Thailand

**Keywords:** Long COVID, respiratory system, adolescent health, well-being, mental health, rehabilitation

## Abstract

**Highlights:**

**Public health relevance—How does this work relate to a public health issue?**

- Long COVID is a public health issue, affecting quality of life and well-being. This study highlights the need for identification and appropriate supportive care to reduce its potential impact in university populations.
- The findings emphasize persistent symptoms, such as fatigue and sleep disturbances, which may impair academic performance and daily functioning.

**Public health significance—Why is this work of significance to public health?**

- This study provides evidence on the impact of Long COVID in a university population, a group often underrepresented in current research.
- Identification of associated factors may contribute to a better understanding of potential impacts and support the development of preventive strategies.

**Public health implications—What are the key implications or messages for practitioners, policy makers and/or researchers in public health?**

- Universities and healthcare providers should support appropriate health monitoring and supportive programs for students experiencing Long COVID symptoms.
- Public health policies should support health monitoring and integrated care approaches to help mitigate potential long-term consequences associated with the pandemic.

**Abstract:**

COVID-19 has had profound global impacts, and Long COVID may continue to affect quality of life and well-being in some individuals. Young adults may be particularly vulnerable to these impacts due to ongoing physiological, behavioral, and psychological development. This study aimed to examine the associations between Long COVID, mental health-related outcomes, and quality of life among university students. A total of 365 Thai undergraduate students participated in this cross-sectional study screening for Long COVID. Long COVID symptoms, mental health, sleep quality, and quality of life were assessed using validated Thai versions of the Long COVID Screening Questionnaire, DASS-21, PSQI, and EQ-5D-5L. Regression and group comparison analyses were conducted between participants with Long COVID and those without Long COVID. Fatigue and cough were the most reported symptoms, while sleep disturbances were also prevalent. Long COVID was associated with significantly lower quality of life scores (*p* = 0.035). However, no significant differences were observed in DASS-21 or PSQI scores between groups. Vaccination doses and prior COVID-19 infections differed significantly between groups (*p* < 0.001 and *p* = 0.017). These findings highlight the multisystem impacts of Long COVID and emphasize the importance of identification and supportive interventions to enhance student health and well-being.

## 1. Introduction

COVID-19 has affected populations worldwide and continues to have substantial long-term health consequences. Long COVID (post-COVID condition) is a chronic condition characterized by persistent or newly developed health problems lasting from weeks to years following an initial SARS-CoV-2 infection. Symptoms are diverse and may affect multiple organ systems [1,2,3]. According to the World Health Organization, Long COVID is defined as a condition in which symptoms typically begin within three months of the initial infection and persist for at least two months. Common manifestations include fatigue, brain fog, and post-exertional malaise (PEM) [1,2,3,4]. The severity of Long COVID remains under-researched, particularly regarding its multisystem effects. Its occurrence and severity may be influenced by factors including vaccination status, infection history, and individual immunological responses. Nevertheless, symptoms can be debilitating and have been associated with increased anxiety, social stigma, and reduced overall quality of life. Long COVID is increasingly recognized as a condition that may lead to significant disability and long-term impairment [4,5,6]. Although the pathophysiological mechanisms underlying Long COVID have not yet been fully elucidated, they have been increasingly studied, and several hypotheses have been proposed, including persistent viral reservoirs or incomplete viral clearance, reactivation of latent viruses, immune dysregulation, autoimmunity, endothelial dysfunction, microbiome dysbiosis, and mitochondrial impairment. These interrelated mechanisms are thought to contribute to persistent inflammation and the multisystem manifestations observed in Long COVID [1,3]. Diagnosis is primarily based on a history of COVID-19 infection and the persistence of symptoms beyond the acute phase. Management focuses on symptom alleviation and improving quality of life through medical care, carefully tailored physical rehabilitation, and supportive therapies [3,7,8].

The long-term health consequences of COVID-19 continue to raise concern, particularly among young adults, who represent a vital segment of the global workforce and future leadership. University students face compounded stressors related to academic demands, lifestyle and educational disruptions, and the prolonged effects of COVID-19 infection [9,10,11]. Mental health problems and sleep disturbances have also been increasingly reported among university students and may be associated with broader pandemic-related factors, potentially affecting academic performance, health, and overall well-being [10,12,13,14,15]. In addition, health risks and unhealthy lifestyle behaviors have long been prevalent in this population. Supporting health screening and monitoring related to the effects of the pandemic may help prevent the progression of these problems among university students. Regarding Long COVID, recent studies have reported that post-COVID-19 symptoms, such as fatigue and cognitive dysfunction may persist for months, impairing daily functioning and learning capacity [16,17]. Long COVID symptoms have also been associated with higher levels of fatigue and behavioral concerns, including increased internet use, among young adult populations [18]. Among the multisystem manifestations of Long COVID and maladaptive health behaviors defined as behaviors that are detrimental to health and may hinder recovery, have emerged as potential concerns within university populations. These combined factors highlight the need to better understand the health and quality of life impacts of Long COVID in this demographic. In the present study, particular focus was placed on mental health-related symptoms and quality of life. Such insight may help inform strategies to promote health, well-being, and sustainable lifestyle behaviors in higher education settings following the COVID-19 public health emergency.

This study aimed to examine whether Long COVID symptoms among Thai university students were associated with life impacts, with a particular focus on mental health, sleep quality, and overall quality of life. It was hypothesized that Long COVID would be associated with adverse impacts on mental health, sleep quality, and quality of life among university students. The findings are intended to help inform the development of supportive care approaches, health promotion initiatives, and academic support strategies to enhance student well-being in the contemporary higher education setting. In addition, data on vaccination status and timing of COVID-19 infection were collected to explore their potential associations with Long COVID in this population.

## 2. Materials and Methods

### 2.1. Study Design and Data Collection

This cross-sectional analytical study employed a quantitative approach using a structured questionnaire to assess research outcomes, including Long COVID symptoms, mental health, sleep quality, and quality of life. The study was designed and reported in accordance with the STROBE guidelines for cross-sectional studies (Table A1). Data were collected between February and March 2025 during the semester break following approval from the institutional ethics committee. Participants were recruited through announcements disseminated via word of mouth and online social media platforms. All participants received an information sheet and provided written informed consent through an active consent process. Individual sessions were conducted to guide participants through the study protocol, and all questionnaires were completed in person by the participants themselves. To reduce potential bias, participant recruitment and data collection were conducted using consistent procedures following the study protocol. All participants completed the same validated Thai-version questionnaires under similar conditions, and in-person administration was used to minimize missing data and improve response completeness.

### 2.2. Participants

Eligible participants were male and female undergraduate students aged 18 to 23 years. All participants were required to understand and communicate in Thai and have a confirmed history of COVID-19 infection verified by either an antigen test kit (ATK) or reverse transcription polymerase chain reaction (RT-PCR) test, regardless of the timing of prior infections. Participants were excluded if they had previously used medications related to sleep regulation or medications known to induce insomnia. Data were collected only from participants who were beyond the acute phase of their most recent COVID-19 infection and had reached at least 5 months after their most recent infection. The sample size was determined using Yamane’s formula with a 95% confidence level and a 5% margin of error, based on an estimated population size of approximately 12,933, resulting in a target sample of 388 participants.

### 2.3. Questionnaires

The research instruments included a Long COVID screening questionnaire developed by the Department of Medical Services, Ministry of Public Health, Nonthaburi, Thailand, based on the World Health Organization definition and international reports. Given that Long COVID has been associated with multisystem physiological involvement and may also be associated with mental health-related outcomes and quality of life, additional instruments were included to capture these domains. Accordingly, the Depression, Anxiety, and Stress Scale (DASS-21), the Pittsburgh Sleep Quality Index (PSQI), and the EQ-5D-5L were used to assess mental health, sleep quality, and health-related quality of life, respectively. All instruments were administered using validated Thai versions. Participants completed the questionnaires within approximately 45 min, and none reported confusion or difficulty understanding the items. Additional health information, including underlying health conditions rather than clinically defined comorbidities, was also self-reported by the participants.

The Long COVID Screening Questionnaire, also known as the COVID-19 Infection and Post-COVID-19 Symptoms Questionnaire for the Public, was used as clinically recommended in Thailand. The questionnaire was designed to identify persistent abnormal symptoms and health consequences following COVID-19 infection, including both general and system-specific manifestations, to support the monitoring and management of individuals with post-COVID-19 conditions. In the present study, the questionnaire was used to identify participants experiencing post-COVID-19 symptoms, and individuals were classified as having Long COVID based on their self-reported responses. The questionnaire acknowledged the World Health Organization definition, which specifies symptoms occurring within three months after infection and persisting for at least two months, while applying a criterion of symptoms lasting more than 4 weeks for screening purposes to identify early post-acute symptoms. The questionnaire assessed symptoms across multiple domains, including general, cardiovascular, neurological, respiratory, psychological, gastrointestinal, dermatological, ear–nose–throat (ENT), musculoskeletal, immune-related, and reproductive symptoms. Participants could also report additional symptoms through an open-ended response option if these were not listed in the questionnaire. Vaccination status, number of COVID-19 infections, and infection severity were also obtained through self-report using the questionnaire. In Thailand, vaccination status can be verified through the official public health application, “MohPrompt”. Based on the questionnaire, COVID-19 severity was classified according to the most severe episode reported by the participant, regardless of the timing of infection. Severity was categorized into four groups according to symptom severity and risk factors: (1) no symptoms, asymptomatic or feeling well; (2) mild, mild symptoms without pneumonia, major comorbidities, or risk factors for severe disease, with normal chest radiographs; (3) moderate, mild symptoms with risk factors for severe disease or major comorbidities, or mild-to-moderate pneumonia without oxygen requirement; and (4) severe, pneumonia requiring oxygen therapy.

The DASS-21 was used to assess levels of depression, anxiety, and stress, providing insight into participants’ overall mental health status. The questionnaire comprises 21 items divided into three subscales, including depression, anxiety, and stress with each containing seven items. Participants rated each statement on a four-point Likert scale ranging from 0 (“Did not apply to me at all”) to 3 (“Applied to me very much or most of the time”), based on their experiences over the past week. The DASS-21 has demonstrated high reliability, with a reliability coefficient (Cronbach’s alpha) ranging from 0.73 to 0.92 for the total scale and its subscales [19].

The PSQI was used to evaluate participants’ sleep quality and disturbances over the past month. This self-administered questionnaire comprises 19 items across seven components which are subjective sleep quality, sleep latency, sleep duration, habitual sleep efficiency, sleep disturbances, use of sleeping medication, and daytime dysfunction. Each component is scored from 0 to 3, with higher scores indicating poorer sleep quality. The sum of the seven component scores produces a global score ranging from 0 to 21. A global score > 5 indicates poor sleep quality, while 5 indicates good sleep quality. Higher scores indicate worse sleep quality. The PSQI demonstrates strong internal consistency, with a Cronbach’s alpha of 0.84 [20].

The EQ-5D-5L was used to assess participants’ perceived quality of life. This standardized questionnaire evaluates five health dimensions consisting of mobility, self-care, usual activities (e.g., work, study, household, family, or leisure activities), pain/discomfort, and anxiety/depression. Each dimension is rated on a five-level scale ranging from 1 (no problems) to 5 (extreme problems or inability to perform activities). Responses were converted into a single index value using a standardized coefficient table, generating a utility score ranging from 0 (worst health state or death) to 1 (best possible health state). The EQ-5D-5L has demonstrated strong reliability, with a reported correlation coefficient of 0.82 [21].

### 2.4. Statistical Analysis

After data collection and verification, all analyses were performed using the Statistical Package for the Social Sciences (IBM SPSS Statistics 28.0) and JASP 0.9 for Windows. Statistical significance was set at *p* < 0.05. Descriptive statistics (mean ± standard deviation) were used to summarize participant characteristics and the prevalence of Long COVID. For inferential analyses, multivariable logistic regression was performed to assess the associations between potential factors and the presence of Long COVID. Regression results were reported as estimates (β), odds ratios (OR), z-values, and Wald statistics. Differences in mental health symptoms, sleep quality, and quality of life between students with and without Long COVID were examined using independent samples *t*-tests or Mann–Whitney *U* tests, depending on data distribution. Effect sizes were reported as Cohen’s *d* for parametric tests and rank-biserial correlation for non-parametric tests.

## 3. Results

Of the 388 students initially recruited, 365 undergraduate students were included in the final analysis. Five students declined participation, and 18 were excluded due to incomplete responses. The mean participant age was 20 years. Approximately 62% of participants were female, and about 10% reported non-severe underlying health conditions. Most participants had received two doses of the COVID-19 vaccine. Approximately half of the participants reported moderate COVID-19 severity, while most of the remaining participants reported mild severity (Table 1).

Among the participants, approximately 7% reported experiencing Long COVID symptoms that met the World Health Organization definition and criteria reported in the international literature, with a sex distribution of 59.26% female and 40.74% male (Table 2). Assessments of mental health, sleep quality, and quality of life among all participants indicated that 42.19% exhibited symptoms of depression, 56.16% reported anxiety symptoms, and 18.08% reported stress. Anxiety was the most prevalent psychological concern, no participants reported extremely severe stress levels. PSQI scores indicated that only 36.16% of participants had good sleep quality. Nevertheless, overall quality of life scores were generally high among the study population (Table 2). Among the overall study population, participants classified as having Long COVID accounted for 3.01% of those reporting depression symptoms, 4.65% of those reporting anxiety symptoms, 0.27% of those reporting stress symptoms, and 0.82% of those with poor sleep quality.

Regarding self-reported Long COVID symptoms obtained from the questionnaire, general manifestations such as fatigue and weakness were commonly reported (Table 3). Among respiratory symptoms, cough was the most frequently observed. Other reported symptom categories included cardiovascular, neurological, gastrointestinal, ENT, musculoskeletal, immune-related, and reproductive symptoms. Notably, among participants with Long COVID, 90% of males reported reproductive symptoms, whereas 31% of females reported irregular menstrual cycles as part of their Long COVID manifestations. Sleep disturbances were also frequently reported, consistent with the broader symptom profile of Long COVID, which may involve both psychological and neurological components (Table 3). No skin-related symptoms (i.e., rashes, hair loss, and skin peeling) or additional symptoms beyond those included in the questionnaire were reported by participants. Although some overlap with pre-existing conditions is possible, only one participant with Long COVID reported an underlying health condition (Table S1).

The number of COVID-19 infections and vaccination doses differed between participants with and without Long COVID (Table S1). Unexpectedly, participants with Long COVID reported a higher number of vaccine doses, which was significantly associated with Long COVID status. However, the temporal sequence between vaccination, infection, and symptom onset could not be determined in the present study. Therefore, this association should be interpreted with caution, as it may have been influenced by reverse causality, recall bias, and unmeasured confounding factors. Long COVID was associated with reduced quality of life, as demonstrated by both regression and group comparison analyses, with affected participants reporting significantly lower EQ-5D-5L scores (Table 4). In contrast, no statistically significant differences were observed in DASS-21 or PSQI scores between groups (Table 4 and Table 5). Nevertheless, the findings suggest potential mental health and sleep-related concerns among participants. The mean PSQI scores were 6.70 in participants with Long COVID and 6.03 in those without Long COVID, indicating a trend toward poorer sleep quality among participants with Long COVID, although this difference did not reach statistical significance. Detailed PSQI outcomes are presented in Supplementary Tables S1–S3.

## 4. Discussion

Considering the impacts of COVID-19 on health behaviors and the higher education system, the present study explored Long COVID symptoms and their associations with mental health-related outcomes and quality of life among university students. Although the prevalence and characteristics of Long COVID may have changed during later phases of the pandemic, persistent post-COVID symptoms continue to be reported worldwide and may still affect well-being and daily functioning in some individuals. The present study showed that approximately 7% of undergraduate students reported experiencing Long COVID symptoms, supporting existing evidence that persistent post-COVID symptoms may continue to affect certain populations [3,5], without directly reflecting the overall burden of Long COVID. Long COVID has been reported worldwide among individuals with prior COVID-19 infection, although prevalence estimates vary considerably across regions, including 35% in Asia, 39% in Europe, 30% in North America, and 51% in South America [22]. The global prevalence of Long COVID remains uncertain due to the lack of standardized diagnostic criteria, inconsistent public health surveillance, and regional differences in pandemic dynamics [22]. These variations may also be attributable to differences in population characteristics, case definitions, and measurement methods across studies. According to Faghy et al., current estimates suggest that between 65 and 400 million individuals worldwide have experienced persistent symptoms following confirmed SARS-CoV-2 infection, with symptoms potentially lasting for one to two years, highlighting the potential long-term impact of this condition [23]. The present study further supports existing evidence that persistent post-COVID symptoms may remain relevant for some individuals and highlights the importance of appropriate follow-up and supportive strategies, particularly among university students and young adults. Based on existing evidence, Long COVID is thought to involve multiple pathophysiological pathways [1,3,24]. Additional mechanisms and hypotheses continue to emerge, reflecting the complexity and heterogeneity of this condition. These observations highlight the need for further mechanistic research, improved diagnostic frameworks, and more rigorously evaluated management approaches [23].

Regarding the Long COVID symptoms reported in this study, fatigue was the most frequently reported symptom (59.3%), followed by cough as the most common respiratory manifestation (51.9%). Sleep disturbances were prevalent, consistent with the psychological domain of Long COVID. Fatigue is a dominant feature of both the acute and convalescent phases of COVID-19, with up to 46% of patients reporting fatigue lasting from weeks to months which is consistent with manifestations of Long COVID [25]. To better characterize post-COVID fatigue, the use of validated screening questionnaires for case detection, standardized interviews encompassing fatigue, mood, and other symptoms, and investigative approaches to identify end-organ damage and mental health conditions have been recommended. In addition, the exclusion of recognized medical and psychiatric causes is necessary [25]. Sleep disturbances may also have multifactorial origins, including both psychological and neurological mechanisms. According to available evidence, the symptom patterns of Long COVID may vary across populations. A large international survey of 11,801 respondents from 33 countries reported chest pain (30%), shortness of breath (27.1%), dysgeusia (25.5%), insomnia (26.7%), muscle or joint pain (24.4%), fatigue (24.2%), and gastrointestinal symptoms (23.3%) [5]. Previous studies have also linked Long COVID to increased psychological and neurological conditions [6,16,17,26]. However, no significant differences were observed in DASS-21 or PSQI scores between participants with and without Long COVID in the present study. Both regression and group comparison analyses indicated that Long COVID was associated with reduced quality of life among undergraduate students. Nevertheless, overall quality of life remained relatively high, suggesting that it is a multidimensional construct not solely determined by Long COVID symptoms. This finding may also reflect that the study population was skewed toward milder cases. Taken together, the present findings are consistent with evidence that Long COVID may affect multiple physiological systems and impair daily functioning and well-being [3,5]. Long COVID symptoms may also exacerbate pre-existing health concerns commonly reported among university students, such as fatigue and sleep disturbances. However, some reported symptoms may represent false positives or reflect underlying conditions, psychological factors, heightened health awareness, or normal convalescence following mild infectious illness [27]. In addition to pharmacological treatments for Long COVID, individualized rehabilitation approaches have shown promise in alleviating symptoms and improving functional recovery in some cases [7,8,28]. Breathing exercises have also been reported to restore basal vascular reactivity and reduce cardiovascular risk among healthcare workers with Long COVID [29]. Although exercise is recognized for its potential benefits in restoring body functions, rehabilitation programs incorporating exercise for individuals with Long COVID should be carefully individualized and may not be appropriate for all Long COVID phenotypes and, in some cases, may potentially cause harmful effects, particularly among individuals experiencing PEM or severe symptoms involving multisystem physiological effects [3,7,8,24]. Therefore, individualized clinical assessment, symptom-based management, careful screening for serious sequelae, and tailored supportive care are important before considering rehabilitation or exercise-based approaches.

Reported risk factors for Long COVID include female sex, a higher number of acute-phase symptoms, early dyspnea, pre-existing psychiatric conditions, and biomarkers such as elevated D-dimer and C-reactive protein (CRP) levels, along with reduced lymphocyte counts [24]. Patients with severe pneumonia have been shown to exhibit the highest cumulative incidence of Long COVID (58.2%), compared with those with mild pneumonia (36.6%) and those without pneumonia (37%) [5]. The risk of developing Long COVID may also be influenced by individual immune responses and viral tissue-targeting patterns, whereby stronger early viral clearance may paradoxically trigger prolonged inflammation and persistent post-infection symptoms [30,31,32]. Previous studies have also reported that Long COVID outcomes may be inversely associated with the number of COVID-19 vaccination doses [33,34]. Nevertheless, substantial heterogeneity across studies has been observed, largely due to inadequate adjustment for potential confounders, such as protective behaviors, as well as issues related to missing data [35]. Regarding the number of infections, previous evidence suggests that the cumulative risk of Long COVID increases with repeated COVID-19 infections [36]. In the present study, the number of COVID-19 infections and vaccination status, rather than infection severity, differed between participants with and without Long COVID. Unexpectedly, participants with Long COVID reported higher numbers of vaccination doses. Regression analysis indicated that only the number of vaccination doses was significantly associated with Long COVID status. However, the temporal sequence between COVID-19 infection, vaccination, and the onset of Long COVID symptoms was not captured in the present study. Consequently, these findings should be interpreted with caution and do not allow conclusions regarding the causal effect of vaccination doses on Long COVID occurrence. Overall, vaccination, particularly booster doses, may provide additional protection against severe COVID-19 and reduce the risk of Long COVID [33,34,35], underscoring the importance of maintaining vaccination strategies, especially in the context of emerging SARS-CoV-2 variants.

This study has several strengths. First, it focuses on university students, a population that remains underrepresented in Long COVID research, thereby contributing to the growing evidence base in young adult populations. Second, validated and widely used instruments were employed to assess study outcomes, enhancing the reliability of the findings. In addition, Long COVID was carefully classified in accordance with the World Health Organization definition and international reports. Third, the study provides a comprehensive assessment of multisystem symptoms and the associations between Long COVID, mental health-related outcomes, and quality of life. Finally, data were collected in person, which may have improved data completeness and reduced missing responses. However, several limitations should be considered. Although the sample size was statistically estimated, it remained relatively small, which may limit the generalizability of the findings. In addition, the number of participants with Long COVID was limited, which may reduce the statistical power of subgroup and comparative analyses and increase the risk of type II error. All participants were recruited from a single setting, which may introduce selection bias and further restrict generalizability. Furthermore, the use of in-person sessions may have excluded certain participant groups, potentially introducing additional selection bias. All data were obtained through self-reported questionnaires, which may be subject to recall bias and response inaccuracies. The identification of Long COVID was based on self-report rather than clinical confirmation. Therefore, self-reported symptoms should be interpreted with caution and may require further screening to improve diagnostic specificity. In addition, the study did not assess whether symptoms occurred as isolated manifestations or in combination. Participants with pre-existing mental health conditions, chronic fatigue, or sleep disturbances were not explicitly excluded, which may introduce residual confounding and influence the observed associations. In addition, unmeasured factors such as lifestyle behaviors, comorbidities, and environmental influences may have affected the results. Importantly, detailed information on the timing of COVID-19 infection and vaccination was not collected, limiting the interpretation of findings, particularly given variations in disease severity across different pandemic phases (e.g., pre-Omicron versus Omicron periods). The study was conducted in a later phase of the pandemic and among young adults, which may influence the prevalence and characteristics of Long COVID. Nevertheless, this population remains important due to the potential impacts on academic performance, health, and well-being.

Given the cross-sectional design, all observed associations should be interpreted as correlational rather than temporal or causal. Therefore, the findings should be interpreted with caution, as they may be influenced by reverse causality and unmeasured confounding factors, while the occurrence of Long COVID is likely shaped by multiple interrelated determinants. The present findings are intended to contribute to the understanding of potential ongoing health-related concerns and student well-being, particularly regarding mental health-related outcomes and quality of life, rather than representing evidence of a broad population-level public health burden. Future research should include larger and more diverse populations and adopt longitudinal and interventional designs. In addition, incorporating both subjective assessments and objective clinical evaluations may improve diagnostic accuracy and enhance understanding of the long-term impacts of Long COVID among university students.

## 5. Conclusions

This study identified potential multisystem effects of Long COVID among university students, particularly sleep disturbances, fatigue, and respiratory symptoms. These findings may help inform future multidisciplinary supportive care and intervention strategies, including appropriate care tailored to individual symptoms and disease manifestations. Promoting positive health behaviors and mental health support within university settings may help mitigate potential long-term consequences associated with COVID-19 and enhance student well-being in contemporary higher education settings.

## Figures and Tables

**Table 1:** Characteristics of participants.

Characteristics (*n* = 365)	Mean (SD) or *n* (%)
Age	20 (1.28)
Sex	
Female	228 (62.47)
Male	137 (37.53)
Underlying health condition	
No	330 (90.41)
Yes	35 (9.59)
COVID-19 vaccination status	
Not received	3 (0.82)
1 dose	22 (6.03)
2 doses	230 (63.01)
3 doses	94 (25.75)
4 doses	13 (3.56)
5 doses	3 (0.82)
Severity of COVID-19 infection	
No symptoms	23 (6.30)
Mild	131 (35.89)
Moderate	194 (53.15)
Severe	17 (4.66)

**Table 2:** Assessment of Long COVID prevalence, mental health (DASS-21), sleep quality (PSQI), and quality of life (EQ-5D-5L) among participants.

Variables (*n* = 365)	Mean (SD) or *n* (%)
Long COVID	
No	338 (92.60)
Yes	27 (7.40)
Dass-21	
Depression	4.15 (3.90)
Normal: 0 to 4	211 (57.81)
Mild: 5 to 6	42 (11.51)
Moderate: 7 to 10	89 (24.38)
Severe: 11 to 13	18 (4.93)
Extremely severe: Above 13	5 (1.37)
Anxiety	4.31 (3.36)
Normal: 0 to 3	160 (43.84)
Mild: 4 to 5	66 (18.08)
Moderate: 6 to 7	79 (21.64)
Severe: 8 to 9	41 (11.23)
Extremely severe: Above 9	19 (5.21)
Stress	4.57 (3.51)
Normal: 0 to 7	299 (81.92)
Mild: 8 to 9	47 (12.88)
Moderate: 10 to 12	13 (3.56)
Severe: 13 to 16	6 (1.64)
Extremely severe: Above 16	0 (0.00)
PSQI	
Good sleep quality: 0 to 5	132 (36.16)
Poor sleep quality: Above 5	233 (63.84)
EQ-5D-5L	0.98 (0.05)

**Table 3:** Distribution of reported Long COVID symptoms among participants.

Long COVID Symptoms (*n* = 27)	*n* (%)
General symptoms:	
Fatigue/laxity	16 (59.26)
Fever	9 (33.33)
Chills	4 (14.81)
Cardiovascular symptoms:	
Palpitations	3 (11.11)
Rapid heartbeat	3 (11.11)
Chest pain	4 (14.81)
Neurological symptoms:	
Loss of smell/taste	10 (37.04)
Headache	7 (25.93)
Dizziness	6 (22.22)
Abnormal movement	4 (14.81)
Memory loss or attention deficit	3 (11.11)
Respiratory symptoms:	
Difficulty breathing/shortness of breath	9 (33.33)
Cough	14 (51.85)
Psychological symptoms:	
Depression	6 (22.22)
Anxiety	4 (14.81)
Sleep problems	8 (29.63)
Gastrointestinal symptoms:	
Diarrhea	4 (14.81)
Nausea and vomiting	5 (18.52)
Abdominal pain	8 (29.63)
Ear, nose, and throat (ENT) symptoms:	
Difficulty swallowing	6 (22.22)
Hearing loss	1 (3.70)
Blurred vision	5 (18.52)
Ear pain	1 (3.70)
Musculoskeletal symptoms:	
Muscle pain	7 (25.93)
Eye, joint, and bone pain	5 (18.52)
Stiff neck	2 (7.41)
Muscle atrophy	0 (0.00)
Acute localized weakness	3 (11.11)
Symptoms of immune system problems:	
Exacerbation of allergic reactions to existing allergies	3 (11.11)
New allergic reactions to previously unexplained allergies	6 (22.22)
Skin blistering (similar to shingles)	0 (0.00)
Reproductive symptoms:	
Female (*n* = 16)	
Irregular menstrual cycle	5 (31.25)
Menopause	0 (0.00)
Male (*n* = 11)	
Testicular pain	10 (90.91)
Sexual dysfunction	10 (90.91)

**Table 4:** Associated risk factors and the impact of Long COVID on mental health (DASS-21), sleep quality (PSQI), quality of life (EQ-5D-5L).

Dependent Variable Long COVID					Wald Test		95% CI (Odds Ratio Scale)	
Coefficients (*n* = 365)	Estimate (β)	Standard Error	Odds Ratio	z	Wald Statistic	*p*-Value	Lower	Upper
COVID-19 vaccination status (dose)	1.282	0.289	3.603	4.430	19.622	<0.001 ***	2.044	6.354
COVID-19 infection (time)	0.361	0.221	1.435	1.635	2.672	0.102	0.931	2.211
DASS-21								
Depression	0.016	0.093	1.017	0.176	0.031	0.860	0.847	1.220
Anxiety	0.021	0.133	1.022	0.160	0.025	0.873	0.787	1.327
Stress	−0.165	0.129	0.848	−1.282	1.645	0.200	0.659	1.091
PSQI	0.138	0.117	1.148	1.177	1.384	0.239	0.912	1.444
EQ-5D-5L	−6.913	3.280	9.945 × 10^−4^	−2.108	4.442	0.035 *	0.000	0.616

* *p* < 0.05; *** *p* < 0.001.

**Table 5:** Comparison of mental health (DASS-21), sleep quality (PSQI), quality of life (EQ-5D-5L) between participants with and without Long COVID.

Variables	Participants with Long COVID (*n* = 27)	Participants Without Long COVID (*n* = 338)	*p*-Value	Effect Size	95% CI	
					Lower	Upper
DASS-21						
Depression	3.30 (4.16)	4.22 (3.88)	0.100	−0.188	−0.394	0.036
Anxiety	3.63 (3.33)	4.36 (3.33)	0.154	−0.164	−0.373	0.061
Stress	3.48 (3.54)	4.66 (3.49)	0.093	0.337	−0.056	0.729
PSQI	6.70 (2.97)	6.03 (1.62)	0.056	−0.384	−0.777	0.009
EQ-5D-5L	0.95 (0.09)	0.98 (0.04)	0.001 **	−0.310	−0.498	−0.093

** *p* < 0.01. For the independent samples *t*-test (stress and PSQI), effect sizes are reported as Cohen’s *d*. For the Mann–Whitney U test, effect sizes are reported as rank-biserial correlation.

## Data Availability

The data that support the findings of this study are available from the corresponding author upon reasonable request.

## Supplementary Materials

The following supporting information can be downloaded at: https://www.mdpi.com/article/10.3390/ijerph23060687/s1. Table S1: Underlying health conditions, COVID-19 vaccination status, number of infections among participants with and without Long COVID; Table S2: Self-reported sleep problems and their impact on daily functioning (PSQI items) among participants; Table S3: Participant responses to the PSQI item assessing difficulty maintaining enthusiasm for daily activities; Table S4: Distribution of responses to the PSQI bed partner or roommate item among participants.

## Abbreviations

The following abbreviations are used in this manuscript:

COVID-19	Coronavirus disease 2019
DASS-21	Depression Anxiety Stress Scale (21 items)
PSQI	Pittsburgh Sleep Quality Index
EQ-5D-5L	EuroQol 5-Dimension 5-Level
SARS-CoV-2	Severe Acute Respiratory Syndrome Coronavirus 2
PEM	Post-Exertional Malaise
ATK	Antigen test kit
RT-PCR	Reverse transcription polymerase chain reaction test
ENT	Ear–nose–throat
CRP	C-reactive protein

## Appendix A

**Table A1:** STROBE checklist.

Section	Item	Description	Page No.
Title	1	Cross-sectional study stated	1–2
Introduction	2–3	Background and objectives	2, 2–3
Methods	4–12	Study design, participants, variables, bias, stats	3–5
Results	13–17	Participants, descriptive data, outcomes	5–11
Discussion	18–21	Interpretation, limitations	11–14
Other	22–23	Funding, ethics	15
