# Supplementary material for: Understanding the Impact of Long COVID on the Lives of Thai University Students
Source: Int J Environ Res Public Health. 2026 May 22;23(6):687. doi: 10.3390/ijerph23060687 (PMC13300268; doi:10.3390/ijerph23060687)
Supplement: Supplementary file 1 [file ijerph-23-00687-s001.zip › ijerph-4283451-supplementary.pdf]

Table S1. Underlying health conditions, COVID-19 vaccination status, number of infections among participants with and without Long COVID

Characteristics	mean (SD) or n (%)		*p*-value	Effect size	95% CI	
	Participants with Long COVID (n = 27)	Participants without Long COVID (n = 338)			Lower	Upper
Underlying health condition	1 (3.70)	34 (10.06)	-	-	-	-
COVID-19 vaccination status (dose)	3 (0.96)	2 (0.61)	< 0.001***	0.461	0.265	0.620
COVID-19 infection (time)	2 (1.05)	1 (0.78)	0.017*	0.221	-0.002	0.423

*$p < 0.05$; ***$p < 0.001$. For the Mann-Whitney U test, effect sizes are reported as rank-biserial correlation.

Table S2. Self-reported sleep problems and their impact on daily functioning (PSQI items) among participants

PSQI (n = 365)	n (%)			
	Not during the past month	Less than once a week	Once or twice a week	Three or more times a week
During the past month, how often have you had trouble sleeping because you…				
a. Cannot get to sleep within 30 minutes	189 (51.78)	116 (31.78)	40 (10.96)	20 (5.48)
b. Wake up in the middle of the night or early morning	223 (61.10)	97 (26.58)	29 (7.95)	16 (4.38)
c. Have to get up to use the bathroom	261 (71.51)	66 (18.08)	30 (8.22)	8 (2.19)
d. Cannot breathe comfortably	273 (74.79)	64 (17.53)	16 (4.38)	12 (3.29)
e. Cough or snore loudly	297 (81.37)	43 (11.78)	16 (4.38)	9 (2.47)
f. Feel too cold	281 (76.99)	78 (21.37)	2 (0.55)	4 (1.10)
g. Feel too hot	291 (79.73)	59 (16.16)	14 (3.84)	1 (0.27)
h. Have bad dreams	279 (76.44)	62 (16.99)	21 (5.75)	3 (0.82)
i. Have pain	290 (79.45)	41 (11.23)	31 (8.49)	3 (0.82)
During the past month, how often have you taken medicine to help you sleep (prescribed or "over the counter")?	365 (100.00)	0 (0.00)	0 (0.00)	0 (0.00)
During the past month, how often have you had trouble staying awake while driving, eating meals, or engaging in social activity?	264 (72.33)	75 (20.55)	17 (4.66)	9 (2.47)

Table S3. Participant responses to the PSQI item assessing difficulty maintaining enthusiasm for daily activities

PSQI (n = 365)	n (%)			
	No problem at all	Only a very slight problem	Somewhat of a problem	A very big problem
During the past month, how much of a problem has it been for you to keep up enough enthusiasm to get things done?	260 (71.23)	86 (23.56)	3 (0.82)	16 (4.38)

Table S4. Distribution of responses to the PSQI bed partner or roommate item among participants

PSQI (n = 365)	n (%)			
	No bed partner or room mate	Partner/room mate in other room	Partner in same room but not same bed	Partner in same bed
Do you have a bed partner or room mate?	286 (78.36)	3 (0.82)	67 (18.36)	9 (2.47)
If you have a room mate or bed partner, ask him/her how often in the past month you have had (n = 79):	Not during the past month	Less than once a week	Once or twice a week	Three or more times a week
a. Loud snoring	40 (50.63)	29 (36.71)	4 (5.06)	6 (7.59)
b. Long pauses between breaths while asleep	75 (94.94)	4 (5.06)	0 (0.00)	0 (0.00)
c. Legs twitching or jerking while you sleep	68 (86.08)	7 (8.86)	3 (3.80)	1 (1.27)
d. Episodes of disorientation or confusion during sleep	68 (86.08)	5 (6.33)	3 (3.80)	3 (3.80)
e. Other restlessness while you sleep	69 (87.34)	5 (6.33)	3 (3.80)	2 (2.53)
